# LncRNA-MIAT-Mediated miR-214-3p Silencing Is Responsible for IL-17 Production and Cardiac Fibrosis in Diabetic Cardiomyopathy

**DOI:** 10.3389/fcell.2020.00243

**Published:** 2020-04-15

**Authors:** Yanqing Qi, Hongyu Wu, Changjiang Mai, Hanqun Lin, Jia Shen, Xiaoyun Zhang, Yakun Gao, Yong Mao, Xupin Xie

**Affiliations:** ^1^Department of Cardiovascular Surgery, Ningbo First Hospital, Ningbo, China; ^2^Department of Vascular Surgery, School of Medicine, Affiliated Hangzhou First People’s Hospital, Zhejiang University, Hangzhou, China

**Keywords:** diabetic cardiomyopathy, IL-17, lncRNA-MIAT, miR-214-3p, fibrosis

## Abstract

As an important complication of diabetes mellitus, diabetic cardiomyopathy (DCM) is characterized by a silent development in its earlier stage and a deficient cardiomyocyte contractility in its late stage. So far, little advance has been achieved to reverse this pathological change. LncRNAs are defined as a large cluster of RNAs without the function of encoding proteins, but have the capacity in controlling gene expression. Interleukin-17 (IL-17), a proinflammatory cytokine, is a key regulator of host inflammation. Clinically, it plays a crucial role in the pathogenesis of cardiac interstitial fibrosis. In this study, we reported that high glucose-induced lncRNA-MIAT upregulation is responsible for proinflammatory IL-17 production in cardiomyocytes. The underlying mechanism is likely due to that lncRNA-MIAT specific attenuates miR-214-3p-mediated inhibitory effect on IL-17 expression. As a result, attenuated IL-17 expression significantly ameliorate cardiac fibrosis, followed by improvement of cardiac contractility. Taken together, our study first suggests that lncRNA-MIAT plays a key role in DCM and targeting lncRNA-MIAT may become a potential strategy to treat DCM.

## Introduction

Diabetic cardiomyopathy (DCM) is mainly characterized by a cardiac dysfunction observed in patients with diabetes mellitus. Generally, DCM occurs in the absence of all other cardiovascular diseases, including coronary artery disease, hypertensive heart disease, valvular heart disease, and congenital heart disease (Wang-Soo and Jaetaek, 2017; Riehle and Bauersachs, 2018; Filardi et al., 2019). Pathologically, diabetes-induced cardiac fibrosis is a central step, causing left ventricular hypertrophy (LVH), diastolic and systolic dysfunction. The mechanism underlying this process are multifactorial and poorly understood. As a result, no treatment is available to prevent or reverse this pathological change (Kawamura et al., 2017; Lorenzo-Almorós et al., 2017; Tate et al., 2019).

Long non-coding RNAs (LncRNAs) are a large cluster of RNAs without the function of encoding proteins, but have the capacity in controlling gene expression. It is well-established that lncRNAs act as key regulators of a variety of physiological process, including cell differentiation and proliferation (Wu et al., 2014; Peng et al., 2016; Xiaoyun et al., 2017). LncRNA myocardial infarction-associated transcript (MIAT) is enriched in endothelial cells, and regulates some endothelial functions that includes migration and vascular sprouting (Cheng et al., 2018). It is reported that lncRNA-MIAT overexpression counteracts the inhibitory effect of miR-22-3p on death-associated protein kinase 2 (DAPK2), a serine/threonine kinase involved in death-inducing pathways. Conversely, lncRNA-MIAT knockdown reduces DAPK2 expression and inhibits apoptosis in cardiomyocytes exposed to high glucose (HG). These results together indicates that lncRNA-MIAT serves as a competing endogenous RNA to upregulate DAPK2 expression by sponging miR-22-3p, as a result, leading to cardiomyocyte apoptosis and subsequent DCM (Xiang et al., 2017). Since the emergence of lncRNAs as regulators of gene expression has shed light on a variety of disease pathogenesis, better understanding of lncRNAs regulatory network will help us uncover the nature of disease further, and ultimately facilitate the improvement of lncRNA-directed diagnostics and therapeutics (Cheng et al., 2019).

Interleukin-17 (IL-17) is a key regulator of host inflammation, which plays a crucial role in the pathogenesis of cardiac interstitial fibrosis. It is reported that ablation of IL-17 alleviates cardiac interstitial fibrosis and enhances cardiac contractility via inhibiting lncRNA-AK081284 in diabetic mice (Zhang et al., 2018). Considering the fact that lncRNA-MIAT is involved in the pathogenesis of DCM and IL-17-mediated cardiac interstitial fibrosis is a major feature of DCM, we questioned if lncRNA-MIAT can regulate IL-17 expression, contributing to the development of cardiac interstitial fibrosis, and ultimately onset of DCM.

For drug discovery, it remains unknown if lncRNAs can regulate a gene expression at a specific locus. Emerging evidence has demonstrated that successful linkage of a specific lncRNA to a functional gene is a prerequisite for providing an alternative to current small molecule approaches for regulating the expression of a specific gene. For example, depletion of metastasis-associated lung adenocarcinoma transcript 1 can downregulate H3K27me3 levels at the E-cadherin promoter, contributing to the acceleration of epithelial-mesenchymal transition (Matsui and Corey, 2016). Taken together, it has become clear that drug development will gain a firmer foundation with the better understanding of lncRNAs.

In this study, we found that HG treatment can upregulate IL-17 production both *in vitro* and *in vivo*. Molecularly, we demonstrated that HG-induced lncRNA-MIAT upregulation is responsible for the production of IL-17. The mechanism is likely due to that lncRNA-MIAT specific attenuates miR-214-3p-mediated inhibitory effect on IL-17 expression. Finally, we demonstrated that knockdown of lncRNA-MIAT in mice significantly improves cardiac function and alleviate cardiac fibrosis. Taken together, our study first suggests that lncRNA-MIAT plays a crucial role in DCM and targeting lncRNA-MIAT may become a potential way to treat the disease.

## Materials and Methods

### Patients Statement

Blood samples from 12 diabetic patients and healthy people were collected at Ningbo First Hospital. None of them has reported a history of hypertension, coronary artery disease or other heart diseases. All written informed consent was obtained from all participants in the study. The documents were kept in the ethics office of Ningbo First Hospital. This study was approved by the Ethics Committee of Ningbo First Hospital.

### Establishment of Diabetic Mice Model

Male C57BL/6 mice were bought from Liaoning Changsheng Biotechnology Co., Ltd. (Liaoning, China). All mice were feed with adaptive food for a week, followed by random allocation into four groups which include control group (Control), diabetic group (DM), diabetes combined with lncRNA-MIAT lentivirus shRNA group (DM + MIAT-shRNA) and diabetes combined with CASP1 inhibitor group (DM + CASP1 I). Mice were administered 150 mg/kg streptozotocin (STZ, Sigma, St. Louis, MO, United States) in citrate buffer (pH = 4.6) once. After 1 week, blood glucose were measured by contour blood glucose meter (Roche, Germany). Successful establishment of diabetes mice was confirmed by testing blood glucose. The mice whose fasting blood glucose (FBG) was above 11.1 mmol/L were considered have diabetes. Diabetic mice were then treated with lncRNA-MIAT lentivirus or CASP1 inhibitor as indicated. LncRNA-MIAT lentiviral shRNA was purchased from Gene Pharma (Gene Pharma, Shanghai, China); 1 × 109 TU lentiviral shRNA was dissolved in 50 μL saline to make the solution. DM + lncRNA-MIAT-shRNA group were given the resulting solution as mentioned.

### Echocardiography

Vevo 1100 high resolution imaging system (Visual Sonics, Toronto, ON, Canada) was used to perform echocardiography on the mice that had received 12-week treatment. Derived left ventricular ejection fraction (EF) and fractional shortening (FS) was measured as previously described (Zhao et al., 2016).

### Transmission Electron Microscopy

The samples were fixed in 2.5% glutaraldehyde (pH = 7.2) and 1% osmium tetroxide as described previously. The slices were processed according to a standard protocol as previously reported (Yu et al., 2017). The images were captured by a transmission electron microscope (JEM-1230, JEOL Ltd., Tokyo, Japan).

### Hematoxylin and Eosin (H&E) and Masson Staining

Mice left ventricular tissues fixed with 4% paraformaldehyde were embedded in paraffin and cut into 5 μm sections. Sections were processed with either H&E or Masson stain kit (Solarbio, Beijing, China) according to the manufacturer’s instructions.

### Immunohistochemistry

The heart samples were fixed by 4% paraformaldehyde followed by embeddedness in paraffin. Sections were stained with primary antibody against IL-17 (1:200) (Cell Signaling Technology, MA, United States), collagen I and collagen III (1:200) (R&D Systems, Minneapolis, MN, United States) overnight at 4°C, respectively. After washing three times with phosphate buffered saline (PBS), the sections were incubated with corresponding secondary antibody at room temperature for 1 h. Diaminobenzidine and neutral gum were used to stain sections. Images were captured by fluorescence microscopy (Nikon 80i, Otawara, Tochigi, Japan). The resulting figures were then processed by Image-Pro Plus 6.0 to quantify the data.

### Cell Culture and Transfection

Primary cardiomyocytes and fibroblasts were extracted from C57BL/6 mice within 3 days of birth. Cells were incubated with 5.5 mM glucose (NG) or 25 mM glucose (HG) for 24 h in Dulbecco’s modified Eagle’s medium (DMEM) supplemented with 10% fetal bovine serum (FBS) (Biological Industries, Beit-Haemek, Israel) at 37°C incubator with 5% CO_2_. Primary cardiomyocytes in HG group were untreated or treated with 100 μM CASP1 inhibitor Ac-YVAD-CMK, according to previous studies (Xiang et al., 2017). Knocking down experiments were performed using X-treme GENE siRNA transfection reagent (Roche, Germany) for small interfering RNA (siRNA) against lncRNA-MIAT (siMIAT), miR-214-3p mimics (miR-214-3p), or anti-miR-214-3p oligonucleotide (AMO-214-3p) with corresponding negative controls (si-NC, NC, AMO-NC), respectively. The interfering RNA sequences were designed and synthesized by RIOBIO (Guangzhou, China) (Table 1). All procedures were performed according to the manufacturer’s instructions.

**TABLE 1:** Interfering RNA sequence.

Interfering		RNA Sequence
miR-214-3p mimic	Forward	5′-ACAGCAGGCACAGAGACCGGCAGU-3′
miR-214-3p mimic	Reverse	3′-UGUCGUCCGUGUCUGUGUCCGUCA-5′
miR-214-3p inhibitor		5′-mAmCmUmGmCmCmUmGmUmCmUm GmUmGmCmCmUmGmCmUmGm UmGmU-3′

## RNA Isolation and Quantitative Real-Time -PCR (qRT-PCR)

TRIzol LS and TRIzol (Invitrogen, Carlsbad, CA, United States) were used to extract total RNA from human serum and mice heart. NanoDrop spectrophotometer (NanoDrop Technologies, Wilmington, DE, United States) was applied to measure RNA concentrations. Reverse transcription was performed using a reverse transcription kit (Toyobo, Japan). The resulting cDNA was amplified for detection by ABI 7500 fast real-time PCR system (Applied Biosystems, CA, United States) using SYBR Green I (Yoyobo, Osaka, Japan). The 2-ΔΔCT calculation was used to determine relative expression levels of the genes. GAPDH was chosen as an internal standard for MIAT and mRNAs. U6 was used as an internal standard for miR-214-3p. The primer sequences for mice samples were listed in Table 2.

**TABLE 2:** PCR primer sequence.

Primer		RNA sequence
GAPDH	Forward	5′-ATCACTGCCACCCAGAAGAC-3′
	Reverse	5′-TTTCTAGACGGCAGGTCAGG-3′
U6	Forward	5′-CTCGCTTCGGCAGCACATATACT-3′
	Reverse	5′-ACGCTTCACGAATTTGCGTGTC-3′
miR-214-3p	Forward	5′-TATACATCAAACAGCAGGCACA-3′
	Reverse	5′-CATTCGATCTTCTCCACAGTCTC-3′
MIAT	Forward	5′-TGGAACAAGTCACGCTCGATT-3′
	Reverse	5′- GGTATCCCAAGGAATGAAGTCTGT-3′
IL-17	Forward	5′-ACCGCAATGAAGACCCTGAT-3′
	Reverse	5′-CAGGATCTCTTGCTGGATGAGA-3′
Collagen-I	Forward	5′-GCCCTTCTGGTCCTATTGG-3′
	Reverse	5′-CTACCAGTGTTGCCAGTGTC-3′
Collagen-III	Forward	5′-CCCCTGGTTCTTCTGGACAT-3′
	Reverse	5′-CCTGACTCTCCATCCTTTCCA-3′

### Western Blotting

Total protein samples were loaded on 10% SDS-PAGE. Proteins were subsequently transferred to nitrocellulose membranes. After blocking with BSA for 2 h at room temperature, the membranes were incubated with primary antibodies against IL-17, collagen-I, collagen-III (1:200) (BIOSS, Beijing, China), or GAPDH (1:1,000) (ZSGB-BIO, Beijing, China), respectively, under rotating conditions overnight at 4°C. The membranes were then washed three times with PBS containing 0.5% Tween 20 (PBS-T), followed by incubation with secondary antibody for 1 h at room temperature. GAPDH was chosen as an internal control. Bands were imaged using GelDox XR system (Bio-Rad, CA, United States). Quantity One software (Bio-Rad) was used to quantify the intensity of the bands.

### Luciferase Assay

To identify if IL-17 and lncRNA-MIAT were direct target of miR-14-3p. Luciferase assay was carried out using HEK293 cell. The 3′UTR of IL-17 and 3′UTR of lncRNA-MIAT were cloned into the downstream of luciferase gene to generate Luc-IL-17-WT and Luc-lncRNA-MIAT-WT, respectively. The 3′UTR of mut-IL-17 and 3′UTR of mut-lncRNA-MIAT were constructed to generate Luc-IL-17-mut and Luc-lncRNA-MIAT-mut vector. For the assay, cells were plated in 24-well plates, followed by either WT or mutant construct with corresponding controls as indicated. Luciferase activity was measured 48 h after transfection using the dual luciferase reporter assay system (Promega, Madison, United States).

### Data Analysis

Each *in vitro* experiment was performed at least three times. Representative images were presented in the Results section. The data were analyzed by SPSS 13.0 software and expressed as mean ± standard deviation (SD). Unpaired Student’s *t*-test and one-way ANOVA analysis were performed to test differences between two groups or among different groups. Two-tailed *P* < 0.05 was considered statistically significant. Graphs were prepared in GraphPad Prism 6.0.

## Results

### LncRNA MIAT and IL-17 Are Overexpressed in the Serum of Diabetic Patients

**FIGURE 1:**
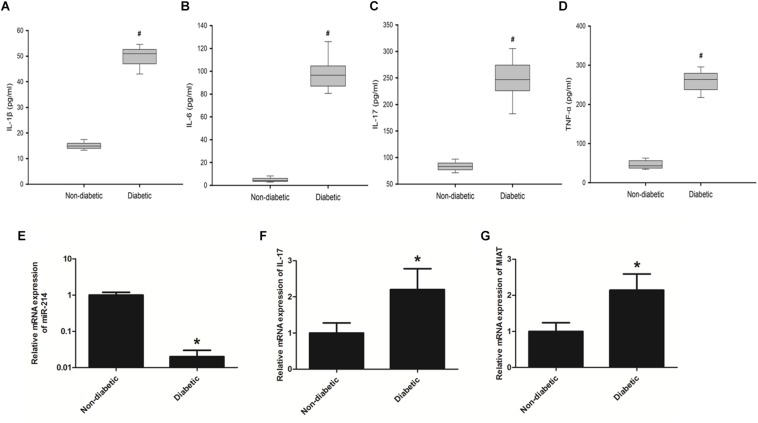
Diabetic patients has elevated expression levels of proinflammatory cytokines as well as lncRNA-MIAT. **(A–D)** Blood samples collected from diabetic patients or healthy controls were processed for ELISA. Protein expression levels of IL-1β, IL-6, IL-17, and TNF-α was measured as above-mentioned, *P < 0.05. **(E–G)** Real-time PCR was performed to analyze the relative mRNA levels of miR-214-3p, IL-17, and lncRNA-MIAT in the serum of either diabetic patients or healthy controls, *p < 0.05, ^#^*p* < 0.01.

Cardiovascular complications are one of the most leading cause of morbidity and mortality in diabetic patients (Uchinaka et al., 2018). The mechanism of DCM is complex, which involves a diversity of pathophysiological changes that include increased oxidative/nitrative stress, activation of various proinflammatory and cell death pathways (De Gonzalo-Calvo et al., 2016; Chen et al., 2019). These changes eventually contribute to cardiomyocyte death and aberrant composition of extracellular matrix with enhanced cardiac fibrosis and increased inflammation (Takayuki et al., 2013; Guanghong et al., 2018). In order to further determine specific factors promoting the onset of DCM by regulating host inflammation, we first performed ELISA to determine protein expression levels of IL-1β, IL-6, IL-17, and TNF-α in the serum of diabetic patients (Supplementary Table S1). The results demonstrated that protein expression levels of IL-1β, IL-6, IL-17, and TNF-α were all significantly increased as compared to those in the serum of healthy controls (Figures 1A–D). Further, we performed qRT-PCR to determine the expression of miR-214-3p, IL-17, and lncRNA-MIAT. The results found that the expression of miR-214-3p was significantly decreased in the serum of diabetic patients (Figure 1E), while gene expression of IL-17 and lncRNA-MIAT significantly upregulated in the serum of diabetic patients as compared to those in the serum of healthy controls (Figures 1F,G).

### HG Treatment Upregulates IL-17 Production in Cardiomyocytes

**FIGURE 2:**
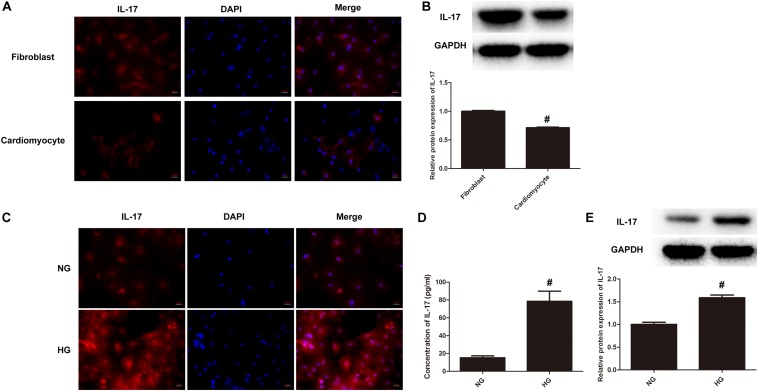
IL-17 production significantly increases upon HG treatment. **(A)** Immunocytochemical staining was conducted using anti-IL-17 (red) in both primary cardiomyocytes and fibroblasts as above-mentioned. Nuclei were counterstained by DAPI (blue). Scale bars, 20 μM. **(B)** Cell lysates collected from both primary cardiomyocytes and fibroblasts were processed for western blotting to determine the protein expression of IL-17. Protein levels of IL-17 were quantitated by densitometric analysis using ImageJ (U.S. National Institutes of Health, Bethesda, MD, United States), normalized to GAPDH. Control group was arbitrarily set a value of 1, ^#^*p* < 0.01. **(C)** Primary cardiomyocytes and fibroblasts were incubated with 5.5 mM glucose (NG) or 25 mM glucose (HG) for 24 h in DMEM supplemented with 10% FBS at 37°C incubator with 5% CO_2_, followed by immunocytochemical staining as above-mentioned. **(D)** Cell supernatants of primary cardiomyocytes and fibroblasts treated with either HG or NG were collected and assayed for IL-17 using ELISA kits, ^#^*p* < 0.01. **(E)** Cell lysates of primary cardiomyocytes and fibroblasts treated with either HG or NG were collected and processed for western blotting to examine the protein expression of IL-17 as above-mentioned, ^#^*p* < 0.01.

It is implicated that IL-17 plays a key role in cardiac ischemia-reperfusion injury and post-myocarditis LV remodeling (Zhang et al., 2018). Considering the fact that DCM shares a similar pathogenesis with ischemic cardiomyopathy, we questioned that the impact of IL-17 on cardiac remodeling of DCM. We first performed confocal immunochemical staining of IL-17 on both cardiomyocytes and fibroblasts. Primary cells generated from C57BL/6 mice were first validated for their origin by confocal microscopy using actin-in and vimentin as two markers (Supplementary Figure S1A). The results showed that IL-17 (Red) is significantly expressed in fibroblasts, as compared to that in cardiomyocytes (Figure 2A). Further, we validated this finding using western blotting. Figures 2B,C demonstrated that protein expression of IL-17 is significantly upregulated in fibroblasts as compared to cariomyocytes, suggesting that IL-17 expression is highly associated with the cell type involved in the inflammation. Next, we determined IL-17 expression between NG group and HG group. Figure 2D demonstrated that IL-17 (Red) is also significantly expressed in HG group, as compared to that in NG group. Similarly, we found that protein expression of IL-17 also significantly increased in cardiac fibroblasts treated with HG as compared to those treated with NG (Figures 2E,F). Taken together, these results indicate that HG treatment may induce cardiac fibroblasts inflammation via production of IL-17.

### Lnc-MIAT Regulates IL-17 Production via miR-214-3p

**FIGURE 3:**
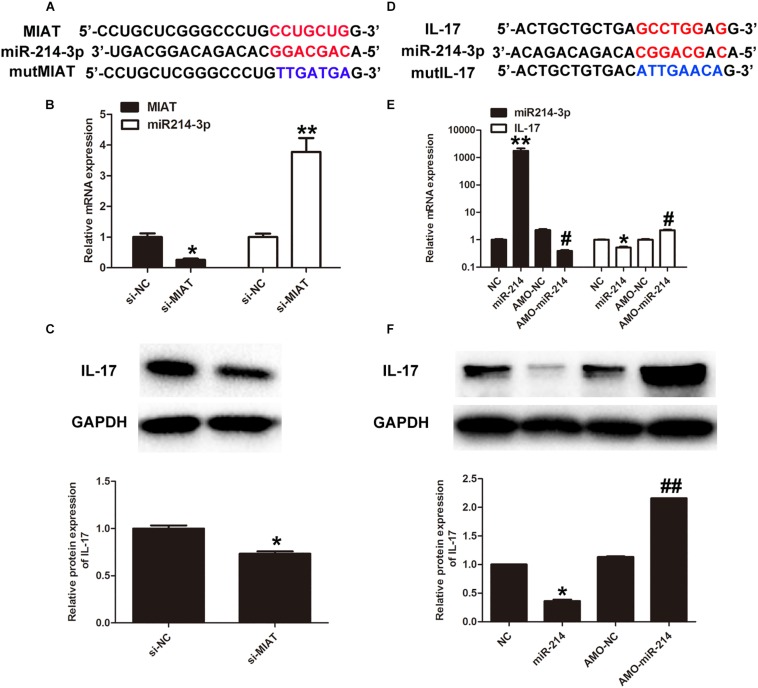
LncRNA-MIAT regulates IL-17 production via miR-214-3p. **(A)** Bioinformatics prediction using miRcode indicated that MIAT sequence contained the putative binding site of miR-124-3p. **(B)** Primary cardiomyocytes were either treated with si-NC (20 μmol/) or si-MIAT (20 μmol/) for 48 h. The expression levels of MIAT and IL-17 expression were detected in cardiomyocytes under either si-NC or si-MIAT group, ^∗^*p* < 0.05. **(C)** Cell lysates of primary cardiomyocytes treated with si-NC or si-MIAT were collected and processed for western blotting to examine the protein expression of IL-17 as Figure 2B mentioned, ^∗^*p* < 0.05. **(D)** IL-17 was predicted as a target gene of miR-124-3p using miRBase. **(E)** Primary cardiomyocytes were treated with NC, miR-214, AMO-NC and AMO-miR-214, respectively, for 48 h. The corresponding expression levels of miR-214-3p and IL-17 expression were detected in cardiomyocytes by q-RT-PCR, ^∗^*p* < 0.05, ^∗∗^*p* < 0.01 (between NC and miR-214); ^#^*p* < 0.05 (between AMO-NC and AMO-miR-214). **(F)** Cell lysates of primary cardiomyocytes treated with NC, miR-214, AMO-NC, and AMO-miR-214, respectively, were collected and processed for western blotting to examine the protein expression of IL-17 as Figure 2B mentioned. Densitometric analysis was also performed as described in Figure 2B, ^∗^*p* < 0.05 (between NC and miR-214); ^##^*p* < 0.01 (between AMO-NC and AMO-miR-214).

Previous study has already found that lncRNA-MIAT was significantly upregulated in diabetic rats with DCM. As a result, high expression of lncRNA-MIAT has been identified as a risk allele for DCM (Xiang et al., 2017). In order to study the mechanism by which lncRNA-MIAT contributes to the onset of DCM, we first used miRcode to predict putative binding sites with specific miRNAs. The results showed that lncRNA-MIAT sequence contained the putative binding site of miR-214-3p (Figure 3A). To verify if the putative binding site of miR-214-3p is correct, we performed luciferase assay. The result showed that miR-214-3p transfection could reduce Luc-IL-17 activity but had no effect on Luc-IL-17-mut activity (Supplementary Figure S2A). Next, primary cardiac fibroblasts were transfected with lncRNA-MIAT-siRNA or Scr-siRNA. The expression of lncRNA-MIAT or IL-17 were evaluate by qRTPCR. The results showed that knocking down lncRNA-MIAT can significantly downregulate gene expression of lncRNA-MIAT, while upregulate the expression of miR-214-3p (Figure 3B). These results suggest that lncRNA-MIAT can negatively regulate the expression of miR-214-3p. Subsequently, we determine if lncRNA-MIAT can regulate the expression of IL-17. Western blotting results demonstrated that knockdown of lncRNA-MIAT significantly decreased the protein expression of IL-17 (Figure 3C), suggesting that lncRNA-MIAT positively regulate IL-17 production. Considering that lncRNA-MIAT regulates both gene expression of miR-214-3p and protein expression of IL-17, we questioned if lncRNA-MIAT regulates IL-17 expression via miR-214-3p. We first performed miRcode to predict putative binding sites between IL-17 and miR-214-3p and found that IL-17 sequence contained the putative binding site of miR-214-3p (Figure 3D). Next, luciferase assay confirmed that miR-214-3p transfection could reduce luc-lncRNA-MIAT activity but had no effect on luc-lncRNA-MIAT-mut activity (Supplementary Figure S2B). To confirm that miR-214-3p can regulate IL-17 expression, we first transfected primary cardiac fibroblasts with either miR-214-3p or AMO-miR-214-3p, followed by extraction of total RNA for qPCR. The results showed that transfection of miR-214-3p significantly decreased gene expression of IL-17, while AMO-miR-214-3p treatment significantly increased mRNA expression of IL-17 (Figure 3E). Further, we performed western blotting to examine protein expression of IL-17 upon either miR-214-3p or AMO-miR-214-3p treatment. Similar results were obtained by demonstrating that miR-214-3p treatment significantly decreased protein expression of IL-17, while AMO-miR-214-3p treatment significantly increased IL-17 production, as compared to corresponding controls (Figure 3F). Taken together, these results suggests that lncRNA-MIAT may regulation IL-17 production via miR-214-3p.

### LncRNA-MIAT-Mediated miR-214-3p Regulates Fibrosis in Cardiac Fibroblasts

**FIGURE 4:**
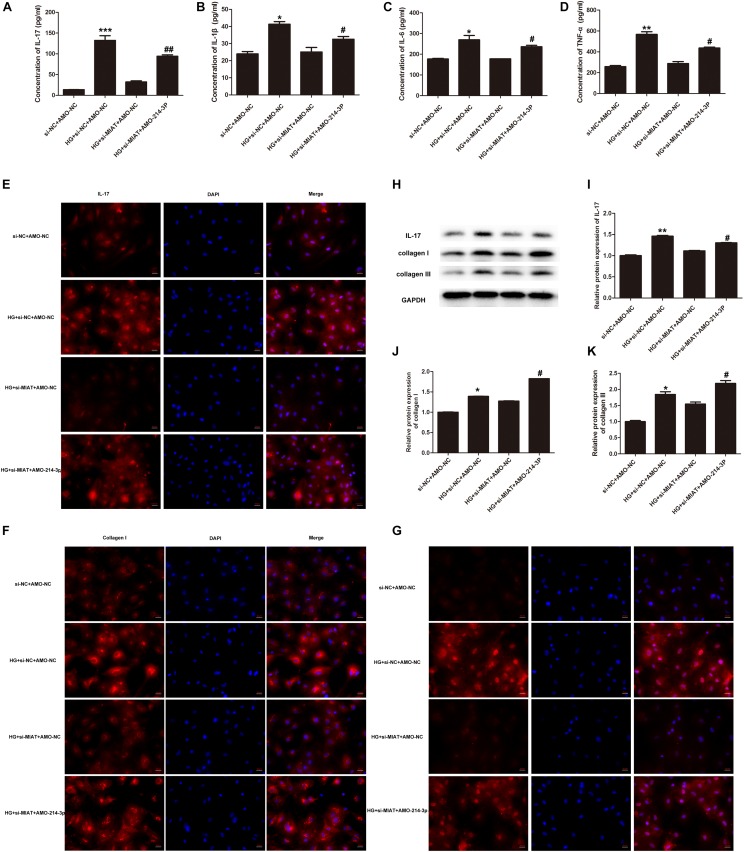
LncRNA-MIAT-mediated miR-214-3p regulates fibrosis in cardiomyocytes. **(A–D)** Cell supernatants were collected from primary cardiomyocytes culture under different treatments as indicated. The protein levels of IL-17, IL-1β, IL-6, and TNF-α were detected using ELISA kits, *p < 0.05, **p < 0.01, ***p < 0.001 (between si-NC + AMO-NC and HG + si-NC + AMO-NC); ^#^*p* < 0.05, ^##^*p* < 0.01 (between HG + si MIAT + AMO-NC and HG + si MIAT + AMO-214-3p). **(E–G)** Immunocytochemical staining was conducted using anti-IL-17 (red), anti-collagen I (red), and anti-collagen III (red), respectively, in primary cardiomyocytes under different treatment groups as above-mentioned. Nuclei were counterstained by DAPI (blue). Scale bars, 20 μM. **(H–K)** Cell lysates of primary cardiomyocytes with above-mentioned treatments were collected and processed for western blotting to examine the protein expression of IL-17, collagen I, and collagen III, respectively, as Figure 2B mentioned. Densitometric analysis was also performed as described in Figure 2B; *p < 0.05, **p < 0.01 (between si-NC + AMO-NC and HG + si-NC + AMO-NC); ^#^*p* < 0.05 (between HG + si MIAT + AMO-NC and HG + si MIAT + AMO-214-3p).

Recent work has revealed a crucial role of inflammation in cardiac repair, remodeling, and fibrosis following myocardial infarction (MI) (Saxena et al., 2016). We questioned if inflammation also plays an important role in the pathogenesis of DCM, which is another type of cardiomyopathy. In order to answer this question, we first examine if HG treatment can drive the expression of a large panel of inflammatory cytokines, including tumor necrosis factor-α (TNF-α), IL-1β, IL-6, and IL-17 in primary cardiomyocytes. The results showed that HG treatment significantly increased the release of TNF-α, IL-1β, IL-6, and IL-17 in the supernatants of primary cardiac fibroblasts (Figures 4A–D). As lncRNA-MIAT is a positive regulator of IL-17, we further determine whether lncRNA-MIAT can also positively regulate other inflammatory cytokines under HG environment. The results demonstrated that knockdowning of lncRNA-MIAT + HG treatment significantly decreased the production of IL-17 as well as that of TNF-α, IL-1β, and IL-6, as compared to HG treatment (Figures 4A–D). Since miR-214-3p is a mediator bridging lncRNA-MIAT and downstream inflammatory cytokines, we further applied AMO-miR-214-3p to evaluate if blockage of miR-214-3p can rescue the production of inflammatory cytokines. The results demonstrated that AMO-miR-214-3p treatment significantly increased the release of proinflammatory cytokines as compared to corresponding control, indicating that lncRNA-MIAT can regulate a panel of inflammatory cytokines via miR-214-3p. Further, we performed confocal immunochemical staining of IL-17 in primary cardiac fibroblasts under different treatment as indicated. The results were similar as what we found in ELISA. Figure 4E showed that HG treatment can significantly increase the protein expression of IL-17 in cardiac fibroblasts, and knockdown of lncRNA-MIAT attenuated the production of IL-17. Moreover, the production can be rescued after AMO-miR-214-3p application (Figure 4E). It is well-documented that the expression of cardiac collagen types I and III is highly associated with cardiac fibrosis, and severe fibrosis impairs cardiac contractility (Uchinaka et al., 2018). Therefore, we next performed confocal immunochemical staining of collagen I and collagen III to determine if HG induces cardiac fibrosis via elevation of collagen I and III. The results demonstrated that HG treatment significantly increased the production of collagen I and collagen III, and the increased production can be inhibited by knockdown of lncRNA-MIAT (Figures 4F,G). We also found that AMO-miR-214-3p treatment can significantly rescue the inhibitory effect of si-MIAT treatment by showing enhanced fluorescence of collagen I and collagen III. Finally, we performed western blotting to examine the protein expression of IL-17, collagen I and collagen III. The results are the same as what we observed by confocal microscopy (Figures 4H–K). Taken together, our results suggest that cardiac fibrosis may be regulated by lncRNA-MIAT-mediated miR-214-3p.

### Ejection Fraction Is Recovered by MIAT Knockdown

**FIGURE 5:**
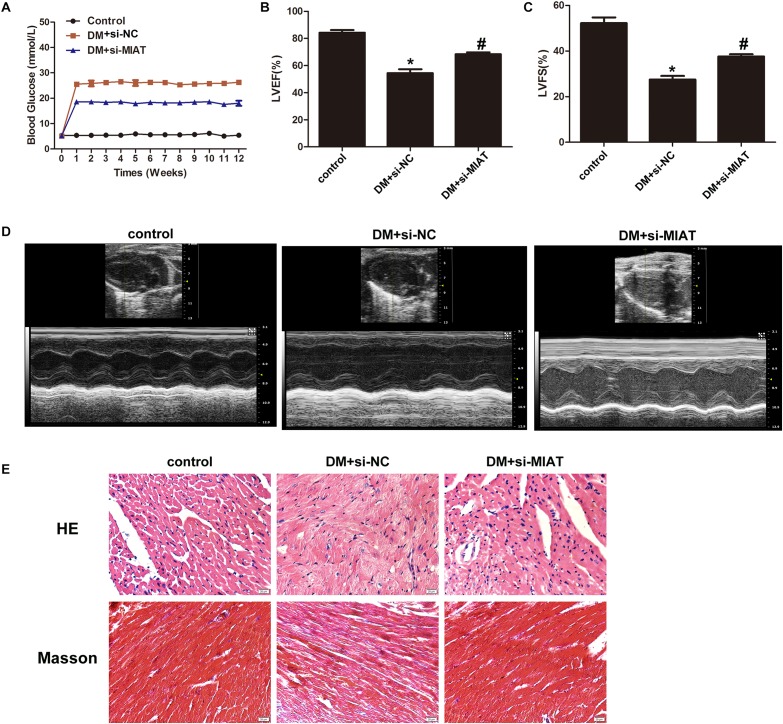
Knockdown of MIAT improves cardiac ejection fraction. **(A)** FBG was tested once a week until 3 month. The FBG curves were plotted among three groups as indicated (*n* = 5 per group). **(B,C)** LVEF% and LVFS% were measured by echocardiography, and the values were plotted as indicated (*n* = 5 per group). *p < 0.05, ^#^*p* < 0.01. **(D)** systolic and diastolic function was evaluated by echocardiography. Representative images were demonstrated as indicated. **(E)** H&E and Masson staining of hearts harvested from control, DM and DM-siMIAT mice at the end of the experiment. Images were taken using the SPOT Insight camera and Nikon ECLIPSE E600 microscope at original magnification ×40. Scale bars, 20 μm.

Since we have found lncRNA-MIAT plays an important role in cardiac inflammation *in vitro*, we next examined the effect of lncRNA-MIAT on cardiac inflammation *in vivo*. First, diabetic mice were successfully established by STZ, followed by injection of lentiviral silenced lncRNA-MIAT (DM + LV-siMIAT) via caudal vein. Successful knock-down of lncRNA-MIAT was confirmed by qRT-PCR (Supplementary Figure S3A). Similar as what we observed in DM patients, the expression level of lncRNA-MIAT in DM mice were significantly increased as compared to healthy mice (Supplementary Figure S3B). Next, we examined if DM + siNC treatment has any effect on blood glucose, LVEF%, LVFS%, cardiac pathological changes and IL-17 expression. The results demonstrated that DM + siNC treatment didn’t change the blood glucose level, LVEF%, LVFS%, cardiac pathological changes nor IL-17 expression, as compared to DM treatment, suggesting that siNC may have little effect on the development of DCM (Supplementary Figures S4A–E). As a result of that, we decided to choose DM + siNC group as a control to both DM + LV-siMIAT group and healthy group. Figure 5A demonstrated a different pattern of blood glucose among three groups, and DM + LV-siMIAT treatment significantly decreased blood glucose as compared to DM + siNC group. However, the blood glucose of DM + LV-siMIAT was still higher than that of control group (Figure 5A). Next, we performed echocardiography to evaluate LVEF% and LVFS% among three groups, respectively. The results showed that DM + LV-siMIAT treatment can significantly rescue the detrimental effect of DM on cardiac contractility by demonstrating a partial recovery of left ventricular ejection fraction (LVEF%), left ventricular fractional shortening (LVFS%) and systolic and diastolic function, suggesting a protective effect of MIAT on cardiac contractility (Figures 5B–D). Further, we performed *H&E* staining of the heart. The results demonstrated that DM + LV-siMIAT treatment partially recover myocardial injury induced by DM, demonstrating a less myocardial lesion and inflammatory cell infiltration pattern (Figure 5E). Similar results were also observed by masson staining, indicating that knockdown of lncRNA-MIAT can protect heart against HG exposure (Figure 5E). Taken together, our results suggest that knockdown of lncRNA-MIAT may serve as a potential strategy for DCM treatment.

### Cardiac Inflammation Is Recovered by Knockdown of lncRNA-MIAT

**FIGURE 6:**
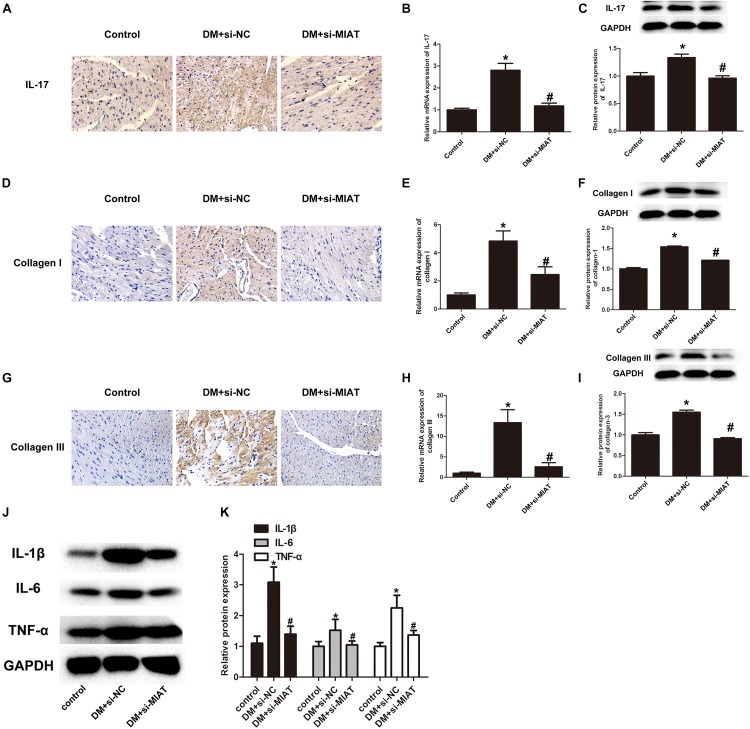
LncRNA-MIAT promote cardiac inflammation in DM mice. **(A,D,G)** IHC staining of IL-17, collagen I, and collagen III in the hearts harvested from control, DM and DM-siMIAT mice at the end of the experiment. The pictures were captured using 100× objective. **(B,E,H)** The expression levels of IL-17, collagen I, and collagen III in the hearts harvested from control, DM and DM-siMIAT mice were detected by q-RT-PCR; *p < 0.05, ^#^*p* < 0.01. **(C,F,I–K)** Tissue homogenate of mice heart with above-mentioned treatments were collected and processed for western blotting to examine the protein expression of IL-17, IL-1β, TNF-α, IL-6, collagen I, and collagen III, respectively, as Figure 2B mentioned. Densitometric analysis was also performed as described in Figure 2B; *p < 0.05, ^#^*p* < 0.01. Since we probed the target protein from the same membrane. The loading control was reused in this figure.

Since we have found that IL-17, collagen I, and collagen III were overexpressed in HG-treated primary cardiomyocytes, we further examine if the same situation can be observed *in vivo*. We first performed IHC to determine IL-17 expression in heart section. The results found that IL-17 expression is significantly upregulated in DM-treated group, as compared to control group, while IL-17 expression is significantly down-regulated in DM + LV-siMIAT group, as compared to DM group (Figure 6A). In order to confirm if the expression of IL-17 is regulated by transcriptional modification, we next perform the qPCR to examine gene expression of IL-17 among three treatment group. The results showed that gene expression of IL-17 is significantly upregulated by DM treatment, while siMIAT treatment downregulates its expression (Figure 6B). The protein expression of IL-17, IL-6, IL-1β, and TNF-α was also evaluated via western blotting. The same results demonstrated that protein expression of IL-17, IL-6, IL-1β, and TNF-α is significantly upregulated by DM treatment, while siMIAT treatment downregulates its protein expression, well corresponding to what we observed *in vitro* (Figures 6C,J,K). According to the same procedure, we next examined the protein expression of collagen I and collagen III. The results showed that the protein expression of collagen I and collagen III were upregulated by DM treatment, while downregulated by siMALT treatment at both transcriptional and translational levels, suggesting that knockdown of MALT is a potential effective strategy in preventing cardiac fibrosis (Figures 6D–I).

## Discussion

LncRNAs are a type of endogenous RNA transcripts longer than 200 nucleotides which epigenetically regulate gene expression but do not have protein-coding potential. To our knowledge, it has found that lncRNAs are aberrantly expressed in DCM, leading to a variety of pathophysiological processes (Pant et al., 2019). LncRNA-MIAT has been first identified to be associated with myocardial infarction in a genome-wide association study in 2006 (Ishii et al., 2006). Since then, aberrant lncRNA-MIAT has been reported to be associated with microvascluar dysfunction, diabetic retinopathy and coronary atherosclerotic heart diseases (Yan et al., 2015; Li et al., 2018; Tan et al., 2019). LncRNA-mediated regulation of gene expression involves a variety of mechanisms that include sequestration of transcription factors, activation of transcription of certain genes, and binding to complementary microRNAs via base pairing to sequester them (Peng et al., 2017; Zhang et al., 2019; Zhou et al., 2019; Wang et al., 2020). Recently, it is well-documented that lncRNAs can influence mRNA levels by interfering with the miRNA pathways, acting as competing endogenous RNAs (ceRNAs). The ceRNAs can inhibit miRNAs through miRNA responsive elements and protect the target mRNAs from repression (Song et al., 2017). This model has been proved to be important in DCM. For example, lncRNA-H19 is involved in myocardial inflammation via regulation of microRNA-675 (Luo et al., 2019); Lnc-kcnq1ot1 could act as a ceRNA for miR-214-3p to regulate the expression of caspase-1 (Yang et al., 2018); And lncRNA-MIAT, serving as a competing endogenous RNA by sponging miR-22-3p regulates the gene expression of death-associated protein kinase-2, which consequently leads to cardiomyocyte apoptosis (Xiang et al., 2017). In our study, we first reported that lncRNA-MIAT, acting as microRNA sponges, attenuates inhibitory effects of miR-214-3p on IL-17 production, leading to cardiac fibrosis in the heart.

It is well-documented that inflammatory response and the production of IL-17 play an important role in several inflammatory diseases that include arteriosclerosis and AMI. *In vivo* studies demonstrated that mice with AMI have elevated levels of IL-17 protein levels as well as IL-1β, inducible nitric oxide synthase (iNOS), IL-6, and matrix metalloproteinase (MMP)-9 genes. Additionally, blockade of the IL-23/IL-17A axis alleviates late post-AMI remodeling. These results together indicate that IL-17 may regulate inflammatory cascade of AMI. Although DCM shares similar clinical outcome as AMI, the pathogenesis of the disease is different from that of AMI. As a result, the functional role of IL-17 in the development of DCM needs to be further explored (Yan et al., 2012). Pathologically, DM-induced cardiac fibrosis is a central step of DCM, which involves complex signaling pathways that leads to remodeling of extracellular matrix (ECM). It is reported that activation of smooth muscle actin (α-SMA), transforming growth factor-β (TGF-β) and reduced functioning of active matrix metalloproteinase-2 (MMP-2) play a crucial role in the development of cardiac fibrosis (Zhen-Guo et al., 2018). IL-17 is a cytokine secreted by T helper 17 (Th17) subset of CD4^+^ T cells. It is known that IL-17 can accelerate the differentiation of myofibroblast by increasing IL-6 production in cardiac fibroblasts. In addition, the neutralization of IL-17 using IL-17 receptor Ad:FC downregulates the expression of IL-6, tumor necrosis factor in the heart, subsequently preventing the progression of DCM (Xie et al., 2013). In our study, we observed that high-glucose treatment significantly upregulates gene expression of IL-1β, IL-6, IL-17, and TNF-α, suggesting a possible mechanism by which IL-17 production together with release of IL-1β and IL-6 contributes to the onset of cardiac fibrosis.

Considering the fact that the expression pattern of lncRNAs is tissue and disease specific, lncRNAs have become superior therapeutic targets for various diseases. By targeting lncRNAs, it can specifically affect cellular subgroups rather than affecting patients systemically as in conventional treatments (Bhan et al., 2017). In our case, we found that knockdown of lnc-MIAT significantly improves heart function *in vivo*, indicating a potential value for further transformation. However, several concerns need to be addressed clearly before going to the next step. First, lncRNAs may have multiple targets. By binding with different targets, lncRNAs may regulate different signaling pathway, leading to different biological outcome; Second, the expression of lncRNA is dynamic-driven, long-term effect of lncRNA regulation still needs to be elucidated; Third, lncRNAs do not only act as transcripts in cis or trans, but can be side products of transcription. Therefore, the analysis of their exact mode of action in disease progression and therapeutic purpose is more complex and difficult than that of protein-coding genes.

In conclusion, our study first reported that HG-induced lncRNA-MIAT is a major reason for DCM. By sponging miR-214-3p, lncRNA-MIAT enhanced the production of IL-17. The emerging IL-17 together with proinflammatory cytokines promotes heart inflammation, eventually leading to cardiac fibrosis and onset of DCM. Our study also shed light on a potential strategy by which targeting lncRNA-MIAT improves heart function and reverse cardiac fibrosis.

## Data Availability Statement

All datasets generated for this study are included in the article/Supplementary Material.

## Ethics Statement

All written informed consent was obtained from all participants in the study. The documents were kept in the ethics office of Ningbo First Hospital. This study was approved by the Ethics Committee of Ningbo First Hospital.

## Author Contributions

YQ and XX designed the studies. CM, JS, HW, HL, XZ, YG, and YM performed the experiments. XX and YQ wrote and revised the manuscript.

## Conflict of Interest

The authors declare that the research was conducted in the absence of any commercial or financial relationships that could be construed as a potential conflict of interest.

## Supplementary Material

FIGURE S1 The identification of cardiomyocyte and cardiac fibroblast. Immunocytochemical staining was conducted using anti-actin-in (red) and anti-vimentin (green), respectively, in primary cardiomyocytes and cardiac fibroblast. Nuclei were counterstained by DAPI (blue). Scale bars, 20 μM.

FIGURE S2 **(A)** The identification of the binding sites between miR-214-3p and IL-17 by luciferase assay. HEK293 cells were transfected with miR-214-3p mimic and luciferase constructs of IL-17 3’UTR or mutant Luc-IL-17-3’ UTR. Luciferase activity was detected 48h after transfection. miR-214-3p inhibitor was treated as previously described. NC and NC inhibitor groups were used as negative controls, ^#^*p* < 0.01; **(B)** The identification of the binding sites between miR-214-3p and lncRNA-MIAT by luciferase assay. HEK293 cells were transfected with miR-214-3p mimic and luciferase constructs of lncRNA-MIAT 3’UTR or mutant Luc-lncRNA-MIAT-3′ UTR as above mentioned, ^#^*p* < 0.01.

FIGURE S3 **(A)** The identification of successful knockdown of lncRNA-MIAT. The expression level of lncRNA-MIAT between DM + siNC and DM + siMIAT were detected, ^∗^*p* < 0.05, ^#^*p* < 0.01. **(B)** The expression of lncRNA-MIAT in diabetic mice. Real-time PCR was performed to analyze the relative mRNA level of lncRNA-MIAT in the serum of either diabetic mice or healthy controls, ^∗^*p* < 0.05, ^#^*p* < 0.01.

FIGURE S4 The difference between DM group and DM + siNC group on cardiac fuction *in vivo*. **(A)** H&E and Masson staining of hearts harvested from control, DM and DM-siNC mice at the end of the experiment. Images were taken using the SPOT Insight camera and Nikon ECLIPSE E600 microscope at original magnification ×40. Scale bars, 20 μm. IHC staining of IL-17 in the hearts harvested from control, DM and DM + siNC mice at the end of the experiment. The pictures were captured using 100 × objective. **(B–D)** LVEF% and LVFS% were measured by echocardiography, and the values were plotted as indicated (*n* = 5 per group). ^#^*p* < 0.01, systolic and diastolic function was evaluated by echocardiography. Representative images were demonstrated as indicated. **(E)** FBG was tested once a week until 3 months. The FBG curves were plotted among three groups as indicated (*n* = 5 per group).

TABLE S1 Clinical information of the patients involved in this study.
