# Supplementary figures and images for: LncRNA-MIAT-Mediated miR-214-3p Silencing Is Responsible for IL-17 Production and Cardiac Fibrosis in Diabetic Cardiomyopathy
Source: Front Cell Dev Biol. 2020 Apr 15;8:243. doi: 10.3389/fcell.2020.00243 (PMC7174588; doi:10.3389/fcell.2020.00243)

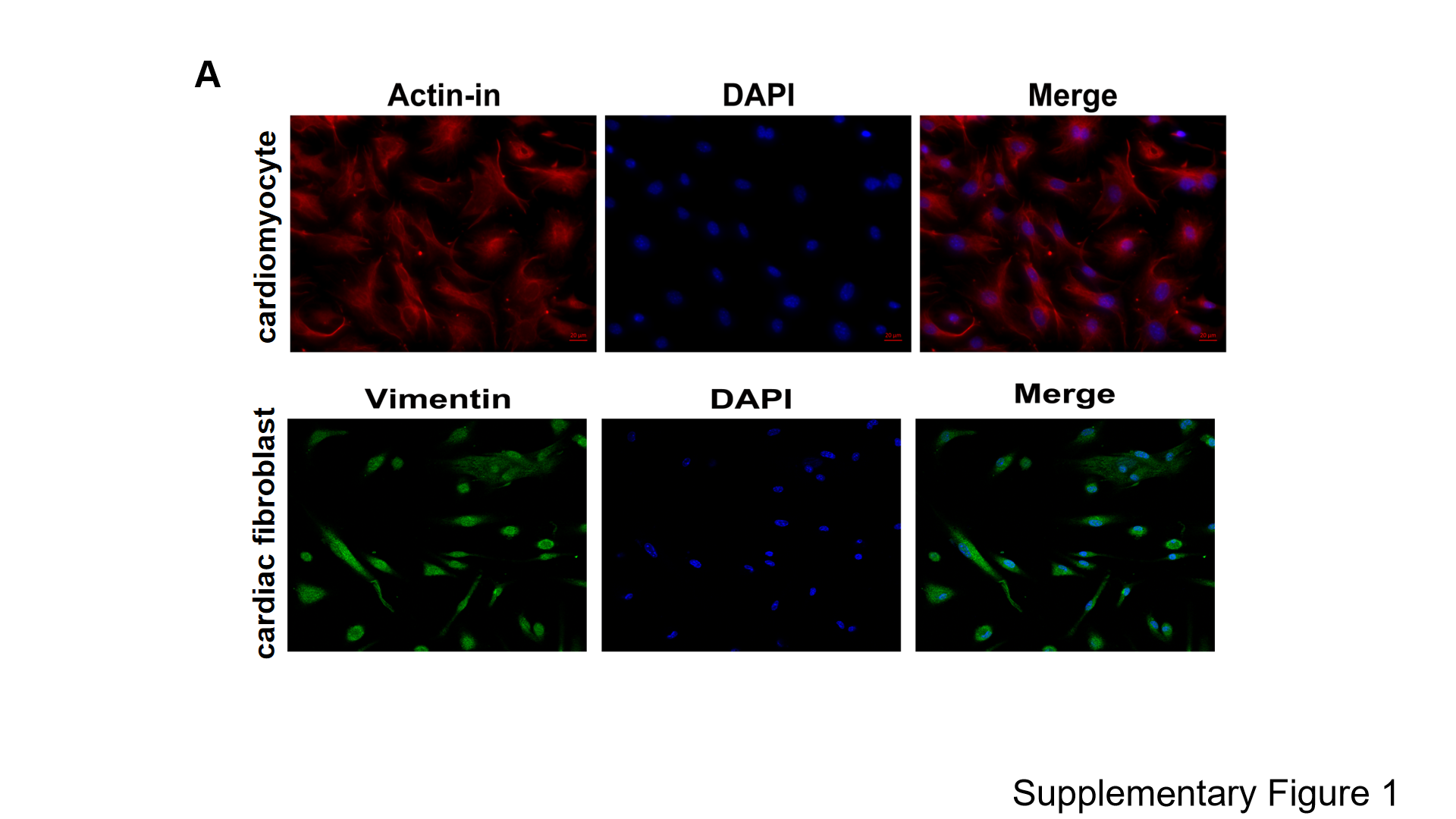

Supplement: FIGURE S1 — The identification of cardiomyocyte and cardiac fibroblast. Immunocytochemical staining was conducted using anti-actin-in (red) and anti-vimentin (green), respectively, in primary cardiomyocytes and cardiac fibroblast. Nuclei were counterstained by DAPI (blue). Scale bars, 20 μM. [file Image_1.TIF]

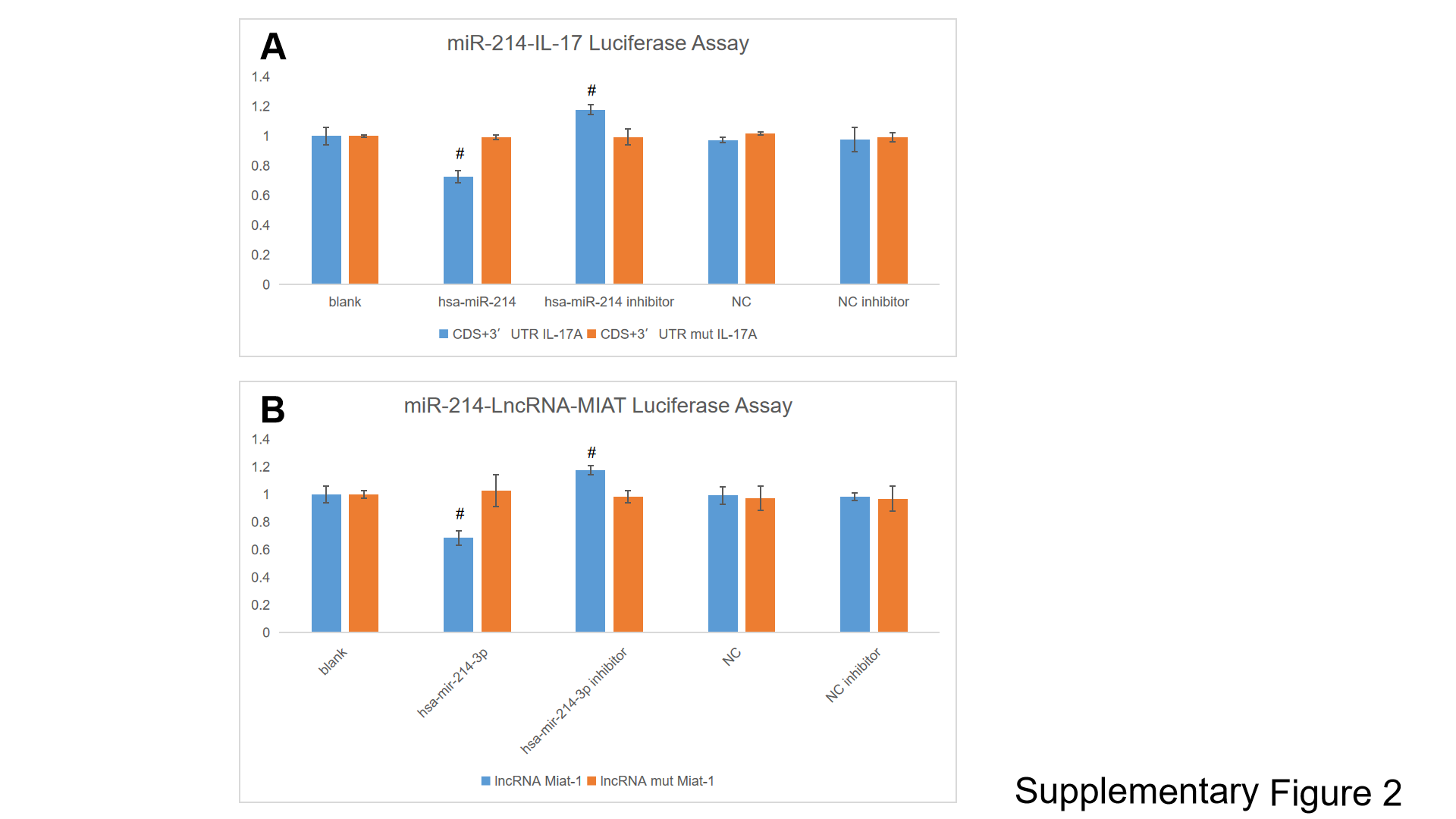

Supplement: FIGURE S2 — (A) The identification of the binding sites between miR-214-3p and IL-17 by luciferase assay. HEK293 cells were transfected with miR-214-3p mimic and luciferase constructs of IL-17 3’UTR or mutant Luc-IL-17-3’ UTR. Luciferase activity was detected 48h after transfection. miR-214-3p inhibitor was treated as previously described. NC and NC inhibitor groups were used as negative controls, #p < 0.01; (B) The identification of the binding sites between miR-214-3p and lncRNA-MIAT by luciferase assay. HEK293 cells were transfected with miR-214-3p mimic and luciferase constructs of lncRNA-MIAT 3’UTR or mutant Luc-lncRNA-MIAT-3′ UTR as above mentioned, #p < 0.01. [file Image_2.TIF]

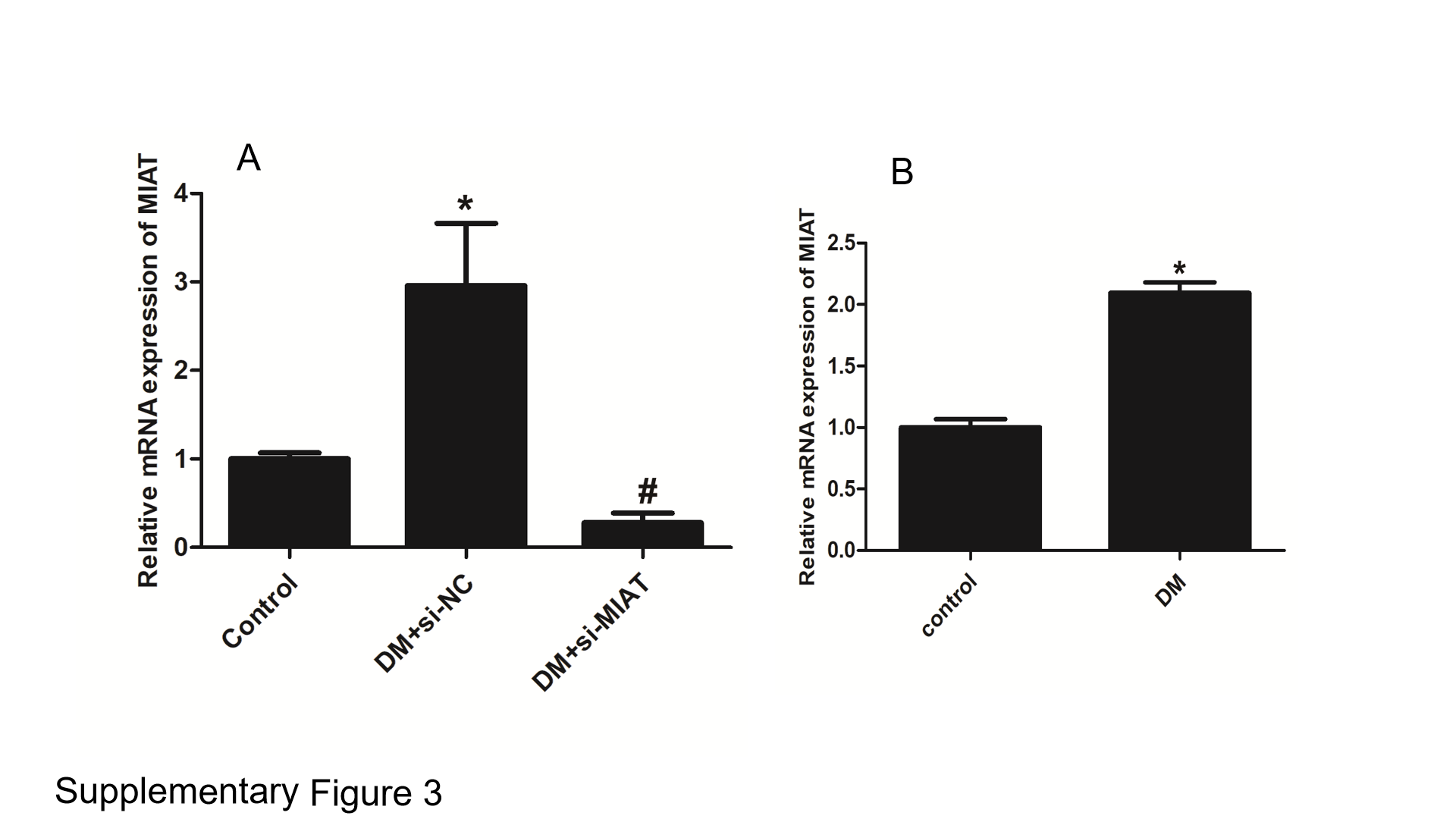

Supplement: FIGURE S3 — (A) The identification of successful knockdown of lncRNA-MIAT. The expression level of lncRNA-MIAT between DM + siNC and DM + siMIAT were detected, ∗p < 0.05, #p < 0.01. (B) The expression of lncRNA-MIAT in diabetic mice. Real-time PCR was performed to analyze the relative mRNA level of lncRNA-MIAT in the serum of either diabetic mice or healthy controls, ∗p < 0.05, #p < 0.01. [file Image_3.TIF]

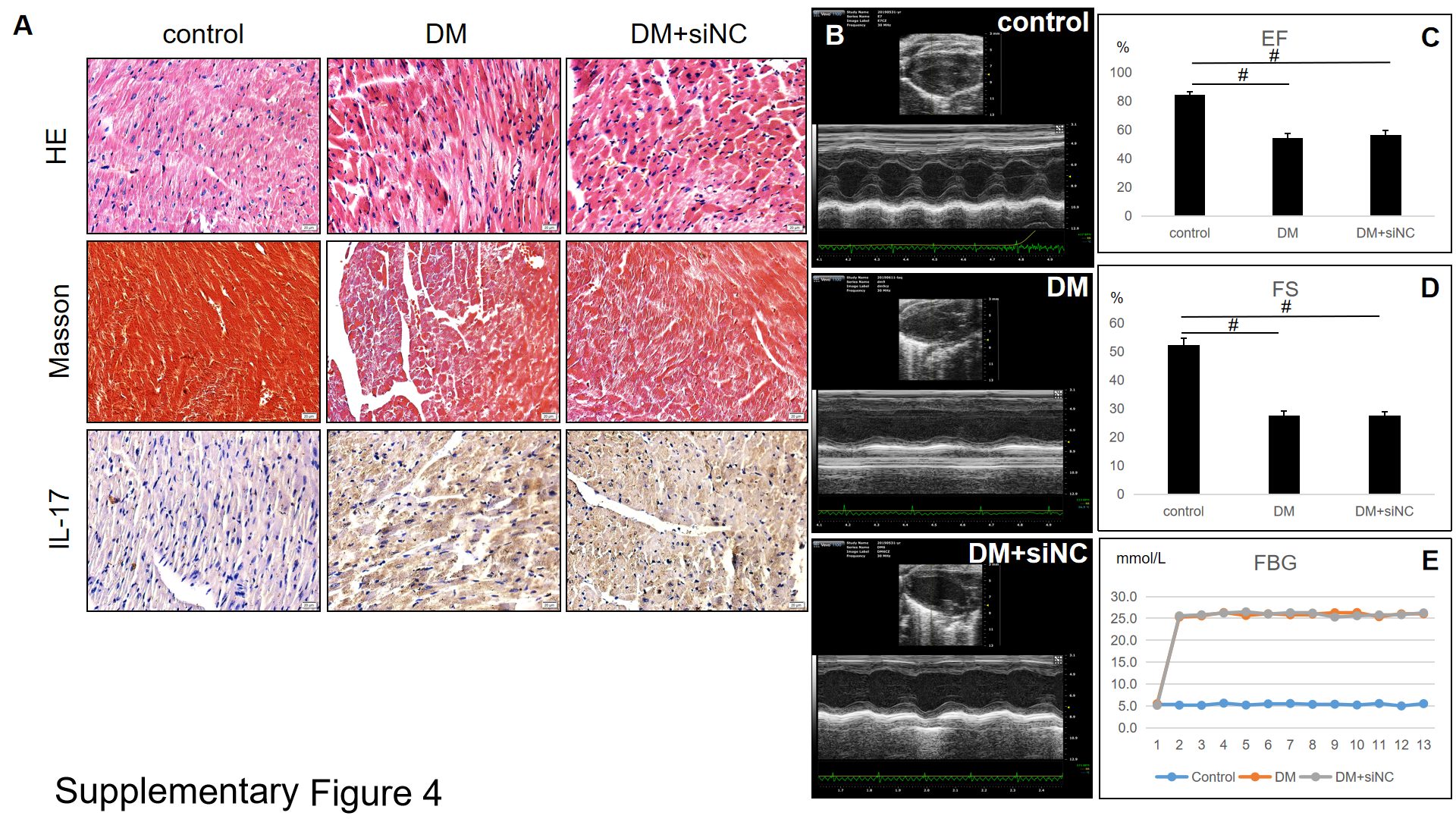

Supplement: FIGURE S4 — The difference between DM group and DM + siNC group on cardiac fuction in vivo. (A) H&E and Masson staining of hearts harvested from control, DM and DM-siNC mice at the end of the experiment. Images were taken using the SPOT Insight camera and Nikon ECLIPSE E600 microscope at original magnification ×40. Scale bars, 20 μm. IHC staining of IL-17 in the hearts harvested from control, DM and DM + siNC mice at the end of the experiment. The pictures were captured using 100 × objective. (B–D) LVEF% and LVFS% were measured by echocardiography, and the values were plotted as indicated (n = 5 per group). #p < 0.01, systolic and diastolic function was evaluated by echocardiography. Representative images were demonstrated as indicated. (E) FBG was tested once a week until 3 months. The FBG curves were plotted among three groups as indicated (n = 5 per group). [file Image_4.TIF]

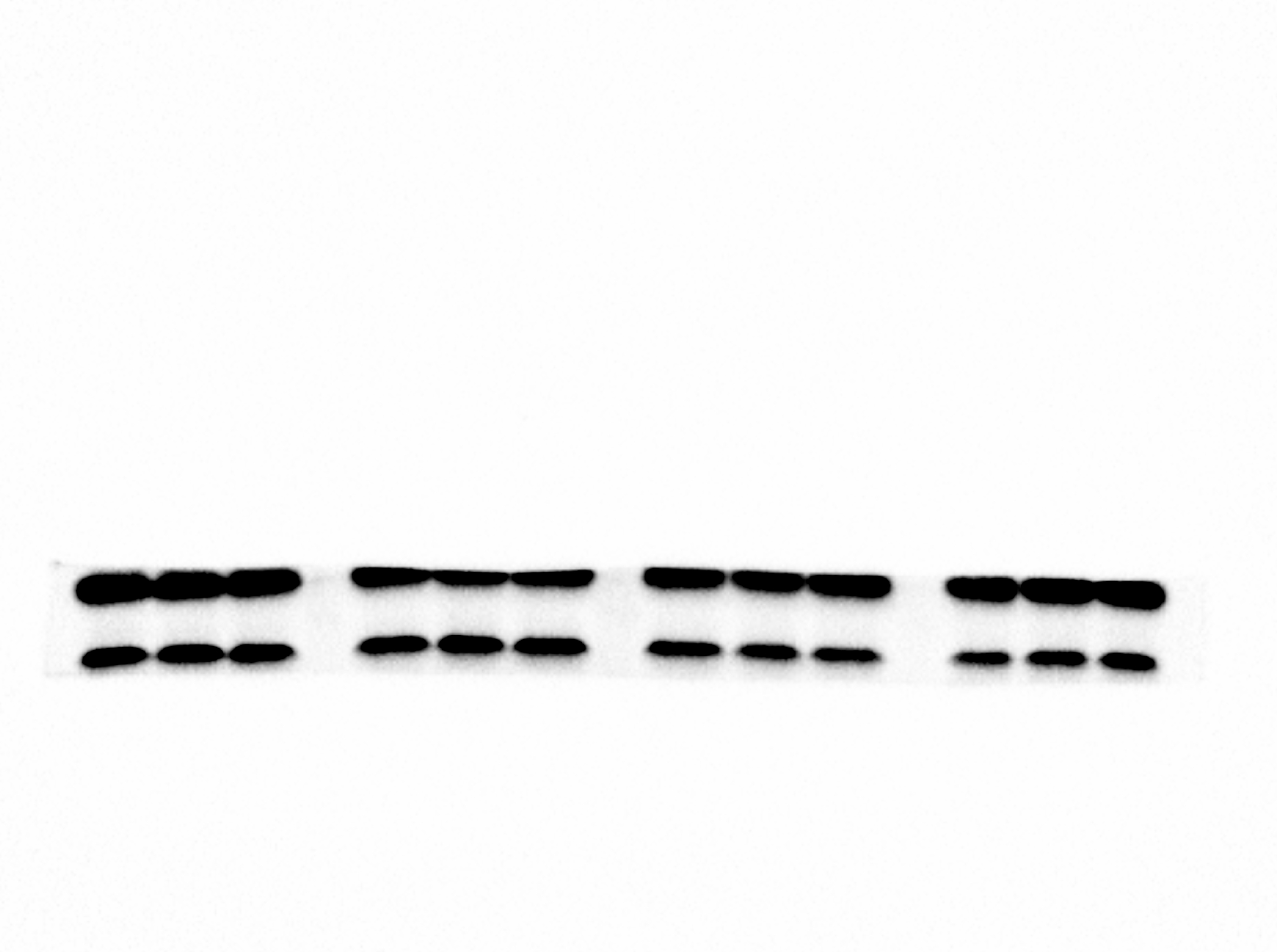

Supplement: Supplementary file 6 [file Data_Sheet_1.ZIP › 1.jpg]

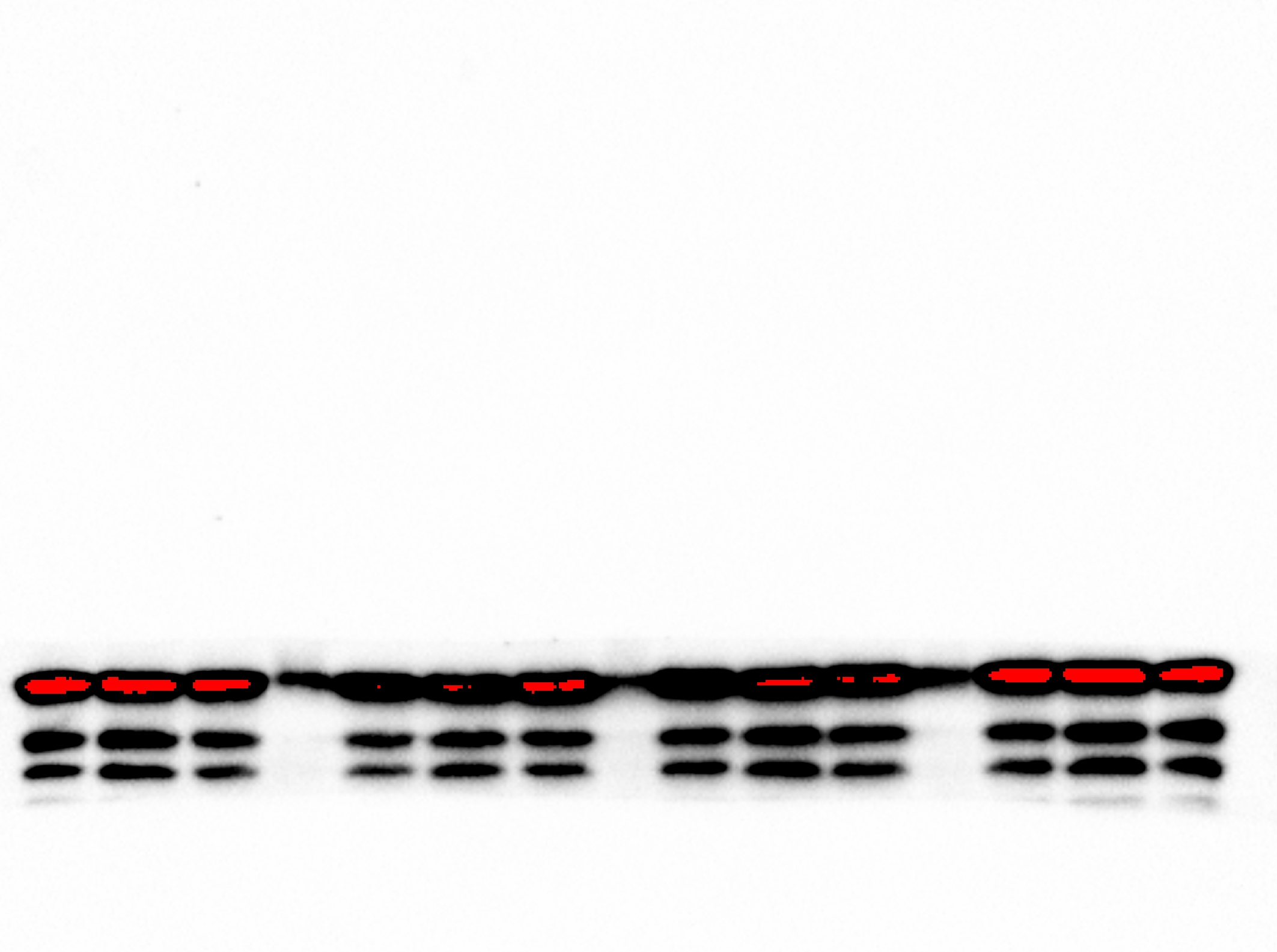

Supplement: Supplementary file 6 [file Data_Sheet_1.ZIP › 2.jpg]

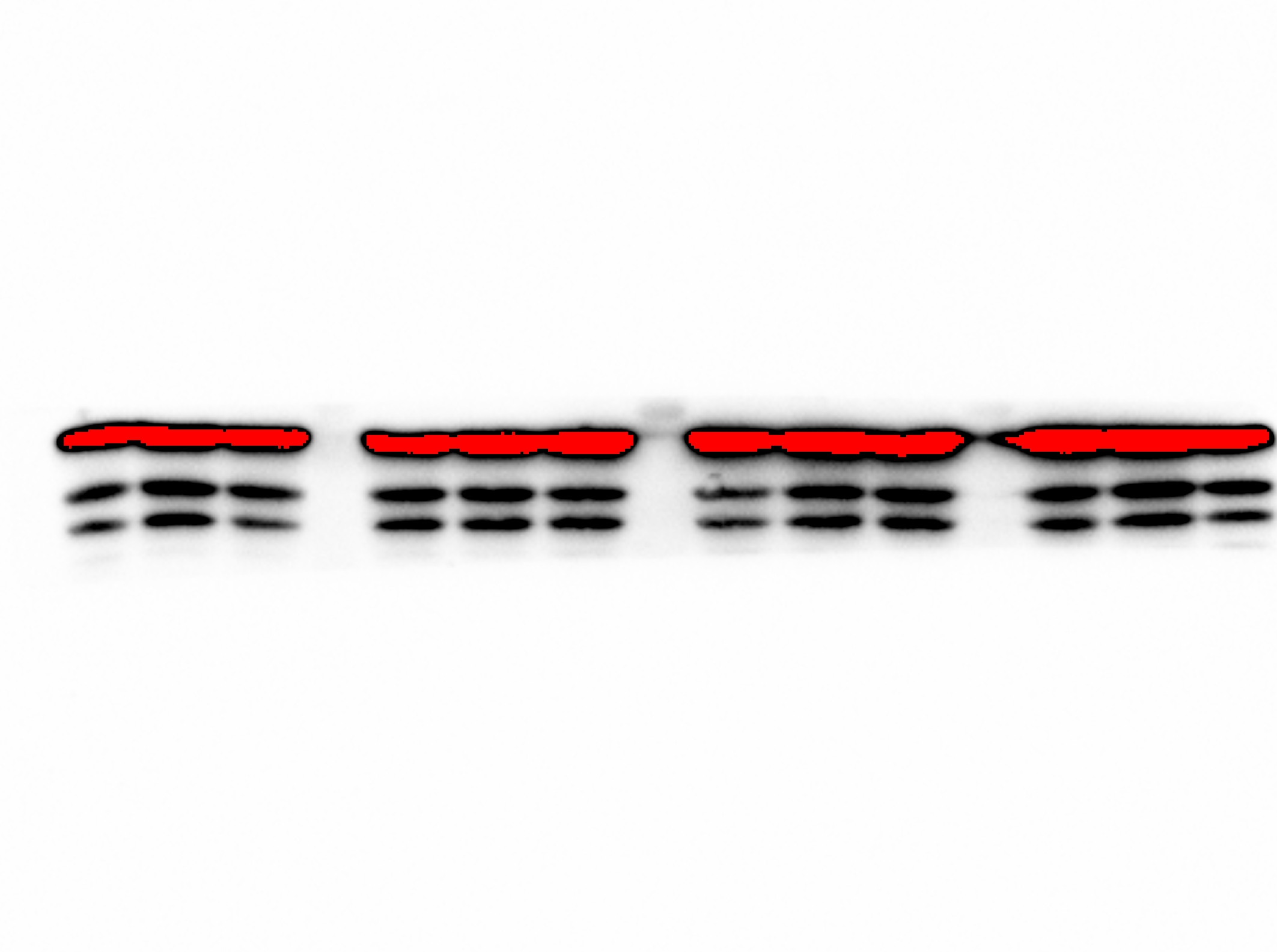

Supplement: Supplementary file 6 [file Data_Sheet_1.ZIP › 3.jpg]

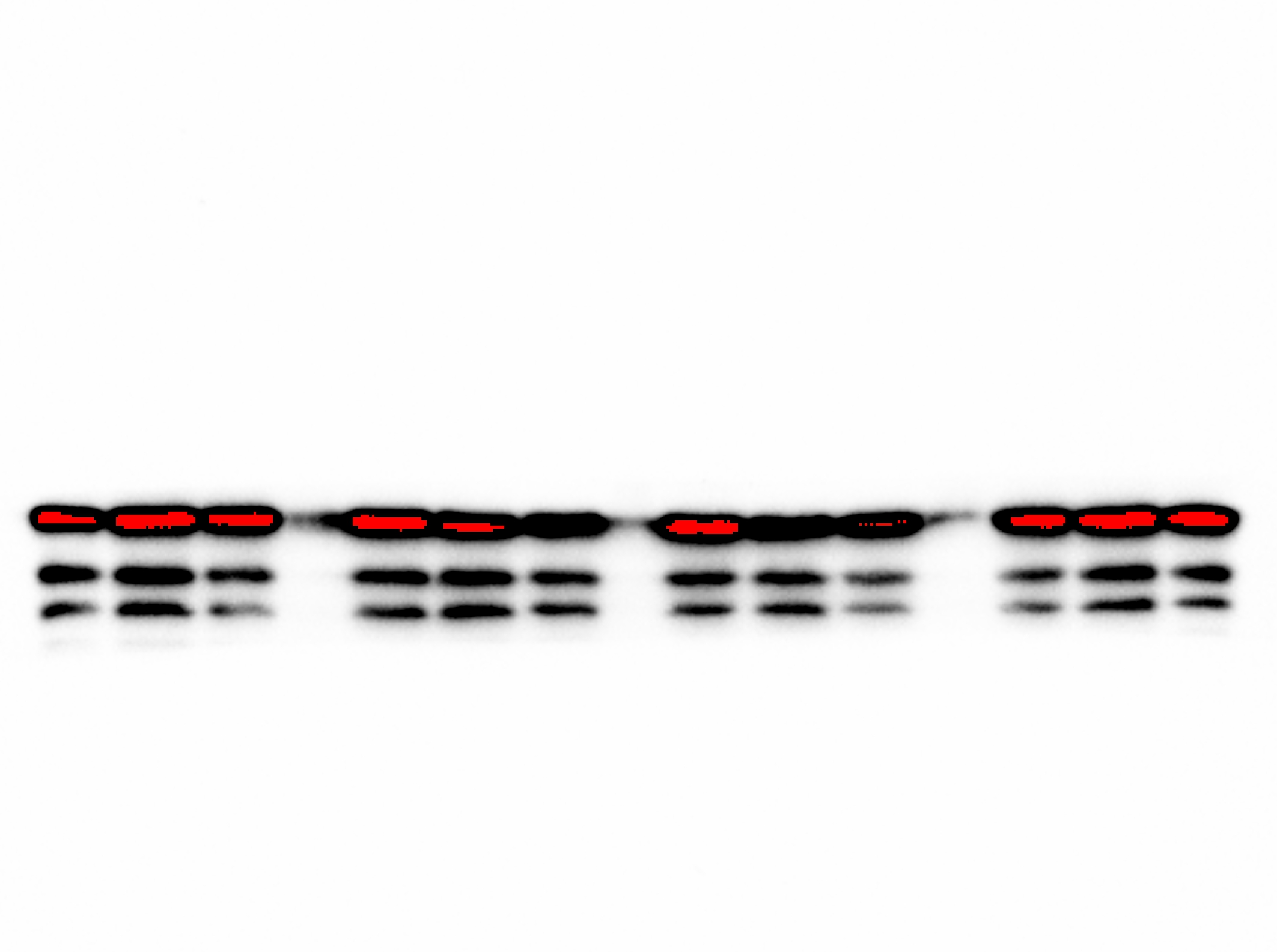

Supplement: Supplementary file 6 [file Data_Sheet_1.ZIP › 4.jpg]

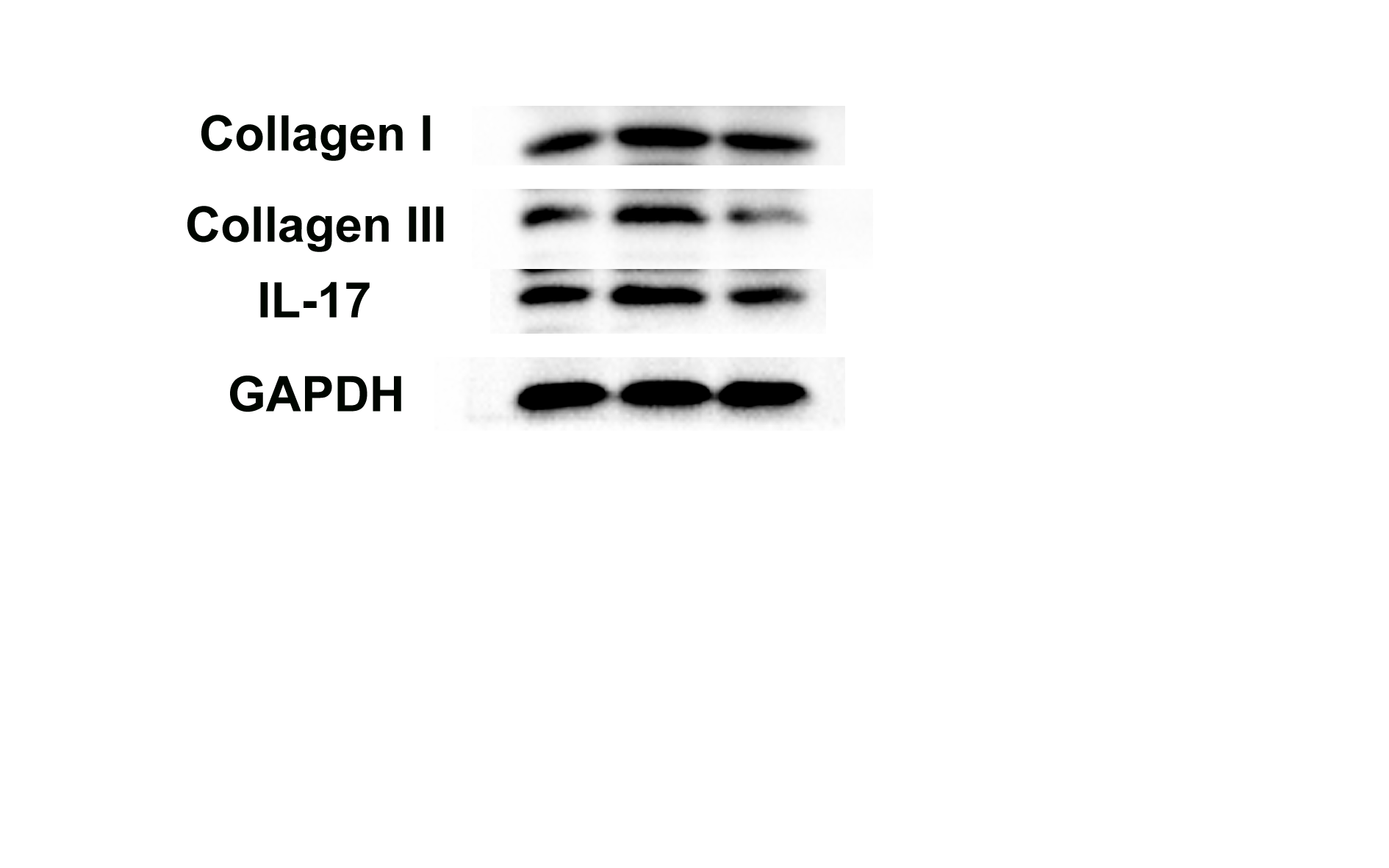

Supplement: Supplementary file 6 [file Data_Sheet_1.ZIP › FIG6E.tif]
